# Relationship Between Incidence of Knee Pain and Ground Reaction Force During Stepping Motion in Older Adults

**DOI:** 10.3390/geriatrics10050126

**Published:** 2025-09-23

**Authors:** Yusuke Oyama, Koki Ishikawa, Toshio Murayama, Tamaki Ohta

**Affiliations:** 1 Faculty of Sports Science, Toin University of Yokohama, Yokohama 225-8503, Japan; 2 Medical Fitness CUORE, Nekoyama Miyao Hospital, Niigata 950-1151, Japan; koki.9756@gmail.com (K.I.); t-ohta@nekoyama.or.jp (T.O.); 3 Faculty of Engineering, Niigata University, Niigata 950-2181, Japan; murayama@ed.niigata-u.ac.jp

**Keywords:** longitudinal study, subjective pain, kinetics, mechanical parameters

## Abstract

**Background:** This 2-year longitudinal study was undertaken to investigate the relationship between incidence of knee pain and ground reaction force (GRF) in stepping motion in older adults. **Methods:** In all, 29 older participants, aged 50 and over (11 males and 18 females; 63.0 ± 6.2 years), presented without knee pain at baseline. The participants performed a 10 s stepping motion at optimal speed on a force plate, and 14 mechanical and temporal parameters of vertical GRF were obtained. Knee pain was evaluated based on subjective complaint during daily activities. The participants were classified into a no pain (NP) group or a knee pain (KP) group. **Results:** Of the 29 participants (11 males, 18 females), 9 (all female) developed knee pain, representing 31.0% of the total participants and comprising the KP group at the follow-up. We compared the amount of change in the evaluated parameters between the two groups and found moderate effect sizes for the mechanical parameters, ΔMshaped (*p* = 0.07, d = 0.77) and ΔF2 (*p* = 0.08, d = 0.72), as well as a flatter change in the bimodal waveform of the GRF in the KP group. **Conclusions:** It was thus suggested that a flattening of the vertical GRF waveform during stepping motion may indicate early biomechanical changes associated with incident knee pain and that waveform changes in GRF may be useful for early detection of functional decline.

## 1. Introduction

Knee pain makes daily activities more difficult and causes a decline in quality of life. Knee osteoarthritis (OA) occurs 22.9% of people aged over 40 years [1]. Factors associated with knee OA include aging, sex, obesity, and mechanical stress on the knee joint [2,3]. A recent study also found that a subset of individuals with advanced radiographic knee OA reported no pain [4], further indicating that radiographic findings alone may not reliably predict symptomatic experience. These findings indicate that subjective knee pain is relatively common in older adults and may occur with or without radiographic OA. Therefore, pain symptoms alone are not sufficient to confirm OA, but may reflect underlying joint stress or early pathological changes.

Early evaluation of the decline in gait function in individuals who have knee pain may prevent progression of knee OA and deterioration in the knee joint. While previous studies [5,6] have reported on the gait characteristics of different grades of knee OA and on the relationship between knee pain and knee adduction moment (KAM) in gait, this has not been adequately investigated, especially in relationship to mild knee pain. Knee OA is thought to begin with knee pain on one side and then to change to a loading pattern across both knees, finally leading to the development of contralateral knee OA. This is thought to be due to mechanical stress on the knee joint [2], which is a factor associated with knee OA, and mechanical stress on one knee joint may be increase when there is a lateral imbalance in a periodic movement, such as gait. Gustafson et al. [7] confirmed that a long hours of gait increases knee joint stiffness on the affected side, placing more stress on the contralateral knee joint. In addition, the ground reaction force (GRF) in gait is generally the gold standard parameter [8]. It has been reported that a bimodal waveform appears in the GRF during gait, where the group with knee pain had a more flatter bimodal waveform than the control group [9]. Therefore, it is important to focus on changes in GRF for the detection of early signs of knee pain and knee OA.

The stepping test among the measures of dynamic balance that are safer than gait movement. The dynamic balance seen in the stepping test is evaluated with parameters such as the number of steps over a certain period of time, double-leg support time and single-leg support time, and differences from the defined tempo [10,11,12]. It has also recently become possible to identify functional independence and frailty using the amount of knee movement seen in stepping tests [13,14]. However, few studies have evaluated stepping motion from a kinetic point of view such as GRF. A previous study confirmed the reliability of GRF measurements during stepping motion and reported that a steady state is reached in fewer steps than in gait [15]. Although stepping differs from gait in movement patterns, it also produces a bimodal vertical GRF waveform, reflecting similar weight acceptance and push-off phases. As a safer and less demanding task for older adults, stepping motion offers a practical alternative to gait for detecting early biomechanical changes related to knee pain. Costello et al. [9] reported that individuals with knee pain exhibit a flatter bimodal vertical GRF waveform during gait. Furthermore, Bjornsen et al. [16] identified sustained GRF loading patterns as a characteristic not only of patients with knee OA but also of those at risk for developing the condition. Given the similarity between gait and stepping GRF waveforms and the lower physical demand of stepping, GRF analysis during stepping motion may be sensitive enough to detect early biomechanical changes associated with knee pain.

Thus, this study investigated the relationship between the incidence of knee pain and GRF in stepping motion in older adults, using a longitudinal study taking place over 2 years. The hypothesis of this study was that the GRFs of those who have developed knee pain will have a flatter bimodal waveform and increased symmetry with a smaller GRF on the affected side. In this study, a “flatter bimodal waveform” is defined as a GRF pattern in which the magnitudes of the first (F1) and second (F3) peaks are reduced, and the mid-stance valley (F2) is elevated, resulting in a less pronounced M-shape. This waveform flattening is also reflected in a lower M-shaped index (ΔMshaped).

## 2. Materials and Methods

### 2.1. Participants

**Figure 1:**
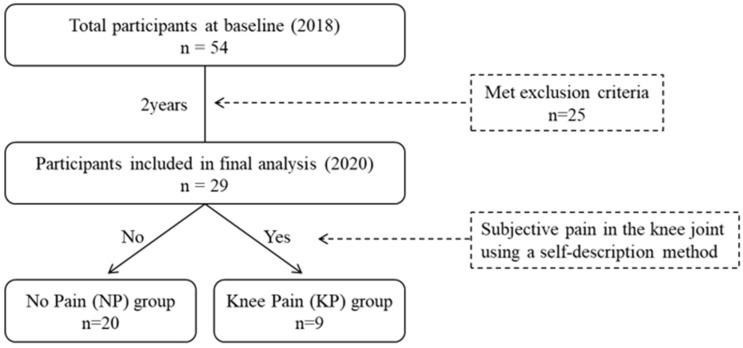
Flowchart of participant selection and group classification.

The participants were older adults aged over 50 years who were members of a designated exercise therapy facility and were not restricted from exercise by a doctor. We included 54 participants (23 men and 31 women) who were part of a baseline survey conducted in 2018 and a follow-up survey conducted in 2020. Inclusion criteria were: (1) age 50 years or older; (2) ability to perform stepping motion independently; and (3) absence of knee pain at the time of the baseline survey. Exclusion criteria were: (1) diagnosis of neurological or musculoskeletal disorders affecting gait; (2) history of knee surgery; and (3) difficulty in understanding or following instructions. Of the 54 participants, 25 were excluded from the final analysis due to knee pain at baseline, loss to follow-up, or meeting exclusion criteria. Among these 29 participants (11 men and 18 women) who did not have knee pain at the baseline survey were analyzed (Figure 1). Their mean age was 63.0 ± 6.2 years, height was 161.3 ± 9.1 cm, and weight 64.7 ± 14.3 kg at the baseline survey. They received sufficient oral and written explanation of the purpose of the study and its measurements before it began. All participants provided written informed consent. The Nekoyama Miyao Hospital Ethics Committee approved this study (approval number: 2017-NR-010, 2020-NR-001). To minimize participant attrition over the two-year follow-up period, we maintained regular communication with participants through exercise therapy facility staff and instructors, as they continued to participate in exercise programs.

### 2.2. Participant Characteristics

The participants were asked about their age, height, weight, and body mass index as well as expressing the degree of subjective pain in the knee joint using a self-description method. Participants were asked to select multiple areas of subjective pain from hip pain, low back pain, knee pain, and ankle pain, and their pain side was also surveyed.

### 2.3. Protocol

Before the measurements were taken, the participants warmed up with a few minutes of stepping and stretching, mainly of the lower limb muscles. They wore comfortable clothing, such as sports training clothes, to avoid interference with the stepping movements, and they were barefoot to minimize external effects on performance.

### 2.4. GRF in Stepping Motion

The participants performed 10 s of the stepping test on a force plate (BM-220, TANITA Co., Ltd., Tokyo, Japan). The force plate used in this study has been modified to record the GRF in the vertical direction at a sampling frequency of 80 Hz on each side by performing a stepping motion. This test was performed on two main trials per participant, basis; however, one practice trial was performed in advance. The practice trial was conducted immediately prior to the main trial and was intended to familiarize participants with the experimental environment. The speed of the stepping motion was set to the self-selected speed, and the examiner instructed the participants to step at the same speed and with the same form as that which they usually used when walking. The stepping test conducted at a self-selected speed has been demonstrated to evaluate disability [13] and frailty [14] in older adults. As these functions are closely linked to dynamic balance ability [17], this study also utilized the stepping test at a self-selected speed. The number of steps in the stepping movement differed by participant, and no limitations of the movements for the lower and upper limbs were specified.

**Figure 2:**
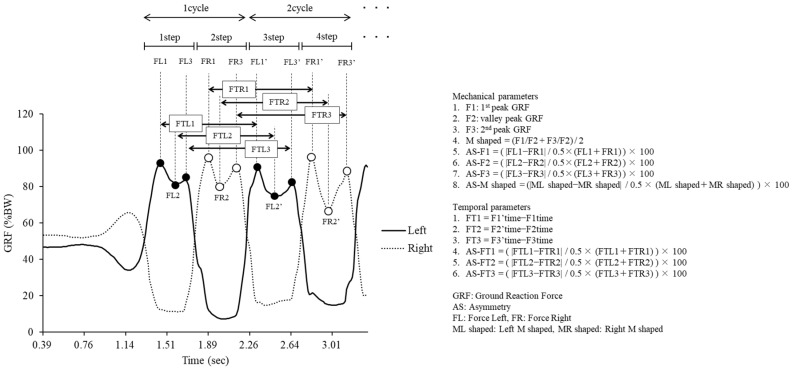
Calculation of mechanical parameters and temporal parameters by the GRF waveform.

The GRF in stepping motion has a bimodal waveform [15]. The extreme values of the two main peaks and the valley during the stepping motion were defined as the value of the first peak (F1), the valley (F2), and the second peak (F3). These values were standardized by body weight. One cycle showed the stepping motion from contact to the following contact of the ipsilateral foot and analysis was later conducted from the third step [15], which was the steady state. The GRF parameters were based on previous studies [15,18] investigating gait and stepping motion and included 8 mechanical parameters showing confirmed reliability between trials (F1, F2, F3, M shaped, AS-F1, AS-F2, AS-F3, and AS-M shaped) and six time parameters (FT1, FT2, FT3, AS-FT1, AS-FT2, and AS-FT3), for a total of 14 parameters (Figure 2). M shaped is considered to draw a clear bimodal waveform with a smaller value, and symmetry is considered to have a smaller lateral difference when the value is closer to 0. In this study, flattening of the GRF waveform was defined as a decrease in F1 and F3, an increase in F2, and a smaller M shaped value, representing a reduction in bimodal sharpness. FT showed the time between F1 and F3 in two consecutive cycles on the left and right. Where the value was small, the tempo of the stepping motion was fast.

### 2.5. Data Analysis

The no pain (NP) group was defined as those who had not develop knee pain by the time of the follow-up survey, and the knee pain (KP) group included those who had developed new knee pain. The 2-year change was calculated by subtracting the baseline survey measurements from the follow-up survey measurements, denoted with a Δ. Normality was confirmed using a Shapiro–Wilk test to compare the two groups at baseline. If the data were normal, paired *t*-tests were performed, and if they were not, the Mann–Whitney U-test was performed. The chi-square test was used to compare the nominal scale variables. In addition, analysis of covariance, adjusted for sex and age, was used to between-group comparisons of the change between baseline and after 2 years in the GRF parameters. The effect sizes (measured with Cohen’s d) were calculated to determine the magnitude of the differences in means, where 0.2 was interpreted as small, 0.5 as moderate, and 0.8 as large [19]. All of these analyses were conducted using EZR version 1.63 [20]. The level of statistical significance was set to *p* < 0.05 for all analyses.

## 3. Results

### 3.1. Comparison of Measures at Baseline and Follow-Up Surveys

Figure 1 shows the flowchart of participant selection and group classification. Of the 54 participants who took part in the baseline survey in 2018, 25 were excluded due to meeting exclusion criteria. As a result, 29 participants without knee pain at baseline were included in the final analysis. At the follow-up survey in 2020, nine participants (31.0%) reported new-onset knee pain and were classified into the knee pain (KP) group, while the remaining 20 participants (69.0%) were assigned to the no pain (NP) group based on self-reported knee pain during daily activities.

Table 1 presents the results of the comparison of each measured parameter for the 29 participants in the study at the baseline and follow-up surveys. The mechanical parameter AS-F3 was significantly higher at the follow-up survey, while the temporal parameters FT1, FT2, and FT3 were significantly lower at the follow-up survey.

### 3.2. Comparison of Characteristics and GRF at Baseline and Follow-Up Survey Between Groups

All nine participants in the KP group were female, whereas the NP group included both male and female participants. The mean age of the KP group was slightly higher than that of the NP group, although the difference was not statistically significant. Nine of the 29 participants (31.0%) in the KP group developed knee pain by the follow-up survey. Table 2 and Table 3 present a comparison of characteristics and mechanical and temporal parameters between the NP and KP groups at baseline and follow-up. Significant differences were found between the two groups in terms of sex and height. There were no significant differences between the two groups in the GRF parameter, but a moderate effect size was identified in the mechanical parameter F1. At follow-up, significant differences between the two groups were observed in the mechanical parameters Mshaped and F1, while no significant differences were found in the temporal parameters.

### 3.3. Comparison of Changes in Ground Reaction Force Parameters in the Two Groups

The results of the comparison between the baseline and follow-up study changes in GRF parameters in the two groups are given in Table 4. While there were no significant differences between the two groups in the GRF parameters, moderate effect sizes were identified for the mechanical parameters ΔMshaped and ΔF2.

## 4. Discussion

In this study, a follow-up survey was conducted two years later for those who had not developed knee pain at the baseline survey. They were classified into two groups based on whether or not they developed incident knee pain, and changes in GRF during stepping movements were compared between the two groups. The results showed that the mechanical parameters for GRF ΔMshaped and ΔF2 tended to decrease in the KP group, with incident knee pain at the time of the follow-up study. It was suggested that the bimodal waveform changes in GRFs during stepping motion could be used to predict the incidence of knee pain at an early stage. The two-year follow-up duration was selected based on previous studies [21,22] showing that symptomatic or structural changes in knee health can occur within this period. These findings support the feasibility and appropriateness of the follow-up period in this study.

A study of 3040 Japanese participants aged 23–95 years found that 21.2% of men and 27.3% of women had incident knee pain over a 3-year period [23]. In this study, 9 participants out of 29 (31.0%) who developed incident knee pain were all women, which partially supported previous studies. The factors that were associated with knee OA included age, sex, obesity, and mechanical stress on the knee joint [2,3], and women appear to be more at risk of developing knee pain than men. This could be a result of decreased quadriceps muscle strength [24] and increased perception of pain influenced by hormonal, biological, and psychosocial factors [25].

There was a moderate effect on the mechanical parameter F1 in the KP group at baseline, which was lower than in the NP group. F1 forms the first peak of the bimodal waveform shown in the vertical GRF of gait and stepping motion [15,18], indicating weight acceptance [26]. This peak relates mainly to the impact of the foot on the ground and the subsequent rapid loading of the weight of the body onto the foot. Since GRF amplitude, particularly F1, is known to be sensitive to stepping tempo [27], the slower baseline tempo observed in the KP group may have contributed to the reduced F1. While the stepping motion was performed at an optimal speed for each participant, tempo differences between groups cannot be ruled out as a confounding factor. However, it is also possible that the lower F1 reflects an early compensatory unloading strategy prior to the onset of pain. This altered loading pattern may serve as a biomechanical precursor to knee pain development, supporting the hypothesis that GRF characteristics could signal early functional decline. Previous studies [28,29] have found reported that a higher KAM at baseline during activities of daily living, such as gait and chair standing, was associated with the development of knee pain in the future. While this study did not evaluate KAM, it is possible that other movement patterns were already present in the baseline survey.

Moderate effect sizes were identified between the two groups in the changes in the mechanical parameters ΔMshaped and ΔF2, with the KP group showing a greater reduction than the NP group. As the Mshaped pattern evaluates the overall bimodal waveform, and the F2 pattern indicates a mid-stance valley [18], it can be assumed that the KP group developed a more flatter bimodal waveform relative to baseline study. Previous studies [9] showed that the vertical GRF in gait has a flatter bimodal waveform in the presence of knee pain or knee OA. In particular, the first and second peaks decreased and the valley between the peaks increased. This is due to a decrease in gait speed [27], and changes in movement patterns [30] may be a factor. The results of this study indicated that ΔT1–ΔT3 increased by the same magnitude in both groups, making it unlikely that the waveform would change to having a slow bimodal waveform due to a decrease in the tempo of the stepping motion. Although the overall stepping tempo was faster at follow-up, the comparable increase in ΔT1–ΔT3 across both groups suggests that tempo change alone does not explain the group differences in GRF waveforms. Therefore, the observed flattening of the bimodal waveform in the KP group is more likely attributable to altered movement patterns associated with knee pain development, rather than changes in stepping speed. A typical gait characteristic of those who have knee pain and knee OA is high KAM [31]. Because vertical GRF is among the most important components of KAM [32], the association between them is significant. In this study, the incidence of knee pain could also feature reduced loading on the affected side to reduce KAM. Barela et al. [33] analyzed vertical GRF during gait using a partial body weight support system, finding that greater support led to a more flat waveform. Kim et al. [30] also found that the vertical GRF in gait showed a more flatter bimodal waveform in the older adults than in the young, which suggests that the gait pattern was chosen to reduce stress on the musculoskeletal system. Thus, it is suggested that the KP group may have taken on a flatter bimodal waveform as a result of compensatory movements developed due to the incidence of knee pain.

These findings may also have important clinical implications. Based on the observed changes in vertical GRF waveforms, particularly the flattening of the bimodal pattern in the KP group, this study suggests that subtle alterations in force patterns during stepping motion may serve as early functional indicators of knee pain development. Unlike radiographic imaging or pain questionnaires, GRF assessment during a simple stepping task is a non-invasive and time-efficient method, making it potentially useful for early screening in both clinical and community settings. Identifying individuals who demonstrate reduced F1 or flatter ΔMshaped and ΔF2 waveforms—prior to reporting overt symptoms—may allow for early intervention strategies. These could include exercise therapy, neuromuscular training, or movement re-education aimed at optimizing load distribution and joint mechanics. Moreover, this approach may help reduce the burden of knee osteoarthritis progression by detecting at-risk individuals before irreversible joint degeneration occurs. Future studies with larger sample sizes should further explore the predictive value of GRF patterns and their integration into preventive screening tools. These findings may have important clinical implications. Monitoring GRF waveforms during a simple, safe stepping task may help identify individuals at risk of knee pain before radiographic signs or symptoms appear. This approach is suitable for community or primary care settings and may enable early interventions—such as exercise therapy or load management—to prevent progression to symptomatic knee OA. Thus, stepping GRF analysis could complement existing assessments to support early detection and timely care in aging populations.

First among the limitations of this study is the likelihood that the individual participant characteristics affected the results. The KP group was small (nine participants) and there was a large bias by sex, which may be reflected in the overall data. The small sample size, especially in the KP group, may have limited the statistical power to detect significant differences, increasing the likelihood of type II errors. To support this, a post hoc power analysis was conducted using G*Power 3.1. Based on the observed effect size (Cohen’s d = 0.77) for ΔMshaped, the total sample size of 29, and α = 0.05, the resulting power was approximately 0.51. This indicates that the study was underpowered to detect moderate effects with conventional significance levels. In addition, given the small sample size, especially in the KP group, only age and sex were included as covariates in the ANCOVA to avoid overfitting and loss of statistical power. These factors were selected due to their established relevance to knee pain and gait mechanics [24,34]. Other potential confounders such as BMI, muscle strength, and physical activity were not included, but should be considered in future studies with larger samples. In addition, the strong sex imbalance (all KP participants were female) may reduce the generalizability of the findings to a broader population. Moreover, the fact that the entire KP group consisted of women may introduce potential bias in the findings, as women may exhibit different biomechanical responses [34], pain perception and hormonal profiles [25,35], compared to men, all of which could influence knee pain progression and GRF patterns. These sex-specific differences in musculoskeletal health could potentially impact the generalizability of the results to male populations. As such, the results should be interpreted with caution and verified in future studies with larger and more balanced samples. In addition, because all participants were recruited from an exercise therapy facility, individuals with severe knee injuries or substantial mobility limitations may have been underrepresented. This potential selection bias could limit the generalizability of the findings to more impaired populations. Second is that the provisions for stepping movements exhibited an influence on the results. In this study, the tempo of the stepping motion was at optimal speed, and therefore, no provisions were made for the tempo or for the contact and takeoff of the foot. These factors may have influenced the GRF values. Third is that the evaluation of knee pain influenced the results of the study. In the study, the participants were classified into groups based on subjective pain alone, so the results for the WOMAC [36] and J-KOOS [37] and the degree of pain were not evaluated in detail. It is also possible that the NP group included some subjects without knee pain who nevertheless have knee OA on radiographs. Costello et al. [9] reported differences in GRF in gait between participants with knee OA only and those with knee pain only. Thus, it is possible that the GRF of the stepping motion will show a marked difference for increased sample sizes and with the participants being classified into groups according to the knee condition. Fourth, although lower limb muscle strength and physical activity level were not directly measured in this study, all participants were regularly engaged in exercise at a designated facility, which may have minimized variability. Furthermore, a recent cross-sectional study [38] examining stepping GRF and muscle strength across age groups demonstrated that their age-related changes do not occur in parallel. This suggests that stepping GRF may reflect neuromuscular or biomechanical characteristics beyond pure muscle strength. Finally, the use of analgesic medications, including over-the-counter pain relievers, was not assessed in this study. Therefore, we cannot rule out the possibility that some participants in either group were using pain medications that may have masked the perception of knee pain. This may have affected the group classification based on subjective reporting, and should be considered when interpreting the findings.

## 5. Conclusions

This study examined the relationship between the incidence of knee pain and GRF in stepping motion in older adults, classifying them into two groups in relation to the incidence of knee pain over a 2-year period. The results indicated that the mechanical parameters of GRF ΔMshaped and ΔF2 tended to decrease in the KP group who developed knee pain during the 2 years, indicating that the waveform altered to form a gradual bimodal waveform. Although these differences did not reach statistical significance, the observed moderate effect sizes imply that changes in the GRF waveform during stepping motion may reflect early biomechanical alterations associated with the development of knee pain. These findings suggest the potential utility of GRF analysis in identifying individuals at risk, but further research with larger samples is needed to validate its predictive value.

## Figures and Tables

**Table 1:** Comparison of measures at baseline and follow-up surveys.

Parameters		Baseline Mean ± SD	Follow-Up Mean ± SD	*p* Value	*ES* (Cohen’s *d*)
Mechanical parameters	Mshaped	0.83 ± 0.06	0.85 ± 0.07	0.20	0.34
	F1	98.7 ± 6.2	96.6 ± 4.9	0.16	0.38
	F2	78.3 ± 4.7	79.1 ± 5.6	0.59	0.14
	F3	91.4 ± 4.4	89.7 ± 3.5	0.12	0.42
	AS-Mshaped	2.7 ± 2.5	2.8 ± 1.9	0.60	0.05
	AS-F1	3.9 ± 2.8	4.8 ± 2.8	0.25	0.31
	AS-F2	4.0 ± 3.3	4.5 ± 3.3	0.64	0.15
	AS-F3	3.5 ± 2.8	5.4 ± 3.6	0.03 *	0.59
Temporal parameters	T1	1.11 ± 0.12	0.86 ± 0.07	<0.001 *	2.61
	T2	1.11 ± 0.12	0.86 ± 0.07	<0.001 *	2.50
	T3	1.11 ± 0.12	0.86 ± 0.07	<0.001 *	2.63
	AS-T1	0.50 ± 0.37	0.41 ± 0.39	0.34	0.24
	AS-T2	0.57 ± 0.59	0.80 ± 1.82	0.06	0.17
	AS-T3	0.44 ± 0.48	0.35 ± 0.26	0.49	0.23

Note. *ES* (Effect Size) 0.20 < *d*: small, 0.50 < *d*: medium, 0.80 < *d*: large. * *p* < 0.05.

**Table 2:** Comparison of stepping motion parameters at baseline survey between groups.

Parameters		NP Group (n = 20) Mean ± SD	KP Group (n = 9) Mean ± SD	*p* Value	*ES* (Cohen’s *d*)
Characteristics	Age (years)	63.2 ± 6.7	64.2 ± 3.8	0.67	0.17
	Sex (male/female)	11/9	0/9	0.01 *	0.52
	Height (cm)	161.5 ± 8.5	152.4 ± 2.3	<0.001 *	1.25
	Weight (kg)	63.6 ± 14.7	59.9 ± 12.2	0.52	0.26
	BMI	24.2 ± 4.2	25.7 ± 4.6	0.40	0.35
Mechanical parameters	Mshaped	0.82 ± 0.06	0.84 ± 0.07	0.44	0.32
	F1	99.9 ± 5.7	96.1 ± 6.7	0.13	0.63
	F2	78.5 ± 4.5	78.1 ± 5.3	0.59	0.08
	F3	91.9 ± 4.2	90.1 ± 4.7	0.33	0.40
	AS-Mshaped	2.8 ± 2.6	2.7 ± 2.4	1.00	0.04
	AS-F1	3.7 ± 2.8	4.4 ± 2.9	0.53	0.25
	AS-F2	4.0 ± 3.1	4.1 ± 3.8	0.99	0.007
	AS-F3	3.1 ± 2.1	4.2 ± 4.0	0.46	0.39
Temporal parameters	T1	1.12 ± 0.13	1.09 ± 0.09	0.50	0.27
	T2	1.12 ± 0.13	1.09 ± 0.09	0.56	0.24
	T3	1.12 ± 0.13	1.09 ± 0.09	0.45	0.31
	AS-T1	0.46 ± 0.37	0.60 ± 0.39	0.38	0.36
	AS-T2	0.62 ± 0.69	0.48 ± 0.26	0.74	0.23
	AS-T3	0.50 ± 0.55	0.31 ± 0.22	0.49	0.40

Note. *ES* (Effect Size) 0.20 < *d*: small, 0.50 < *d*: medium, 0.80 < *d*: large. * *p* < 0.05.

**Table 3:** Comparison of stepping motion parameters at follow-up survey between groups.

Parameters		NP Group (n = 20) Mean ± SD	KP Group (n = 9) Mean ± SD	*p* Value	*ES* (Cohen’s *d*)
Characteristics	Age (years)	63.2 ± 6.7	64.2 ± 3.8	0.67	0.17
	Sex (male/female)	11/9	0/9	0.01 *	0.52
	Height (cm)	161.5 ± 8.5	152.4 ± 2.3	<0.001 *	1.25
	Weight (kg)	63.6 ± 15.0	59.6 ± 12.7	0.49	0.28
	BMI	24.2 ± 4.3	25.5 ± 4.8	0.46	0.30
Mechanical parameters	Mshaped	0.83 ± 0.07	0.89 ± 0.04	0.03 *	0.95
	F1	98.0 ± 5.0	93.5 ± 3.0	0.01 *	0.99
	F2	78.0 ± 5.6	81.5 ± 5.0	0.13	0.64
	F3	90.0 ± 3.7	88.9 ± 3.2	0.45	0.31
	AS-Mshaped	2.9 ± 2.2	2.3 ± 1.2	0.44	0.32
	AS-F1	4.3 ± 2.3	5.8 ± 3.8	0.19	0.54
	AS-F2	3.8 ± 3.2	6.1 ± 2.9	0.05	0.74
	AS-F3	4.9 ± 3.7	6.5 ± 3.3	0.28	0.45
Temporal parameters	T1	0.87 ± 0.07	0.83 ± 0.07	0.20	0.53
	T2	0.88 ± 0.07	0.83 ± 0.07	0.14	0.60
	T3	0.87 ± 0.07	0.83 ± 0.07	0.21	0.52
	AS-T1	0.39 ± 0.32	0.46 ± 0.53	0.72	0.18
	AS-T2	0.94 ± 2.17	0.48 ± 0.54	0.33	0.25
	AS-T3	0.33 ± 0.25	0.39 ± 0.29	0.57	0.23

Note. *ES* (Effect Size) 0.20 < *d*: small, 0.50 < *d*: medium, 0.80 < *d*: large. * *p* < 0.05.

**Table 4:** Amount of change in mechanical and temporal parameters.

Parameters		NP Group (n = 20) Mean ± SD	KP Group (n = 9) Mean ± SD	*p* Value	*ES* (Cohen’s *d*)
Mechanical parameters	ΔMshaped	−0.01 ± 0.05	−0.05 ± 0.05	0.07	0.77
	ΔF1	1.90 ± 5.06	2.57 ± 8.46	0.79	0.11
	ΔF2	0.47 ± 5.23	−3.41 ± 5.76	0.08	0.72
	ΔF3	1.87 ± 3.55	1.20 ± 6.67	0.73	0.14
	ΔAS-Mshaped	−0.18 ± 3.14	0.36 ± 2.57	0.66	0.18
	ΔAS-F1	−0.64 ± 3.61	−1.38 ± 5.09	0.66	0.18
	ΔAS-F2	0.22 ± 4.87	−2.05 ± 5.15	0.26	0.46
	ΔAS-F3	−1.80 ± 4.12	−2.29 ± 4.60	0.78	0.12
Temporal parameters	ΔT1	0.25 ± 0.11	0.26 ± 0.10	0.81	0.09
	ΔT2	0.25 ± 0.11	0.26 ± 0.10	0.64	0.09
	ΔT3	0.25 ± 0.11	0.25 ± 0.10	0.91	0.01
	ΔAS-T1	0.07 ± 0.48	0.13 ± 0.38	0.75	0.13
	ΔAS-T2	−0.32 ± 2.35	−0.01 ± 0.57	0.74	0.16
	ΔAS-T3	0.17 ± 0.68	−0.07 ± 0.39	0.42	0.39

Note. *ES* (Effect Size) 0.20 < *d*: small, 0.50 < *d*: medium, 0.80 < *d*: large.

## Data Availability

The data that support the findings of this study are available from the corresponding author upon reasonable request.
